# Supplementary material for: A Likelihood Approach for Real-Time Calibration of Stochastic Compartmental Epidemic Models
Source: PLoS Comput Biol. 2017 Jan 17;13(1):e1005257. doi: 10.1371/journal.pcbi.1005257 (PMC5240920; doi:10.1371/journal.pcbi.1005257)
Supplement: S1 File — (TAR.GZ) [file pcbi.1005257.s014.tar.gz › HSPH_Online-SI-Revision/MSS10/n100-extreme/n100-extreme_NEW2_table0.pdf]

1	newly 3 weeks spec	7253.	7163.51	{7036., 7271.}	1.42024	0.00622933	newly 3 weeks cumul	27162.	27350.9	{27151., 27494.}	0.725462	0.00313496	newly 5 weeks spec	4336.	3911.66	{3822., 3981.}	9.78644	0.0447798	newly 5 weeks cumul	37280.	36633.1	{36417., 36823.}	1.73527	0.0076062	newly 54 weeks cumul	48774.	45907.5	{45743., 46112.}	5.87705	0.0263065
2	newly 3 weeks spec	5965.	5899.33	{5783., 6027.}	1.5207	0.00669677	newly 3 weeks cumul	20994.	20454.6	{20266., 20632.}	2.56926	0.0113251	newly 5 weeks spec	3796.	3952.4	{3866., 4059.}	4.79399	0.0203663	newly 5 weeks cumul	29744.	29293.3	{29083., 29583.}	1.52858	0.00672413	newly 50 weeks cumul	42279.	42989.	{42865., 43440.}	2.48852	0.0107808
3	newly 3 weeks spec	6513.	6175.64	{6064., 6275.}	5.17979	0.0231321	newly 3 weeks cumul	27002.	26086.3	{25884., 26251.}	3.3913	0.0149895	newly 5 weeks spec	3181.	2844.11	{2769., 2921.}	10.5907	0.0487192	newly 5 weeks cumul	34839.	33182.8	{33011., 33359.}	4.75381	0.0211566	newly 55 weeks cumul	41209.	38517.4	{38324., 38697.}	6.53158	0.0293389
4	newly 3 weeks spec	6774.	6779.59	{5736., 7265.}	8.13685	0.0362894	newly 3 weeks cumul	33063.	32992.6	{30397., 34089.}	4.05366	0.0178841	newly 5 weeks spec	2428.	2389.99	{1688., 2726.}	14.3184	0.0673258	newly 5 weeks cumul	39680.	39450.1	{35240., 41118.}	5.30431	0.023625	newly 59 weeks cumul	42886.	42707.1	{37170., 45053.}	6.69092	0.029886
5	newly 3 weeks spec	8431.	7295.65	{7172., 7449.}	13.4664	0.0631172	newly 3 weeks cumul	31964.	29502.6	{29209., 29926.}	7.70041	0.0348903	newly 5 weeks spec	4440.	3228.28	{3150., 3342.}	27.291	0.13923	newly 5 weeks cumul	42439.	37704.5	{37400., 38233.}	11.1559	0.0515232	newly 56 weeks cumul	51326.	43115.9	{42875., 43716.}	15.996	0.0759476
6	newly 3 weeks spec	7676.	7428.77	{5962., 7996.}	7.40138	0.0350089	newly 3 weeks cumul	28666.	28057.7	{24200., 29378.}	5.2646	0.0241732	newly 5 weeks spec	4357.	4149.19	{2739., 4663.}	13.0574	0.0659163	newly 5 weeks cumul	38952.	37821.8	{31055., 40063.}	6.76453	0.0317241	newly 54 weeks cumul	50195.	48428.7	{36052., 52432.}	9.98725	0.0481972
7	newly 3 weeks spec	7326.	4782.15	{4651., 4918.}	34.7236	0.185349	newly 3 weeks cumul	27951.	21482.9	{21214., 21785.}	23.1409	0.11433	newly 5 weeks spec	3953.	1923.62	{1832., 2009.}	51.3377	0.31305	newly 5 weeks cumul	37123.	26469.7	{26116., 26812.}	28.6972	0.146917	newly 52 weeks cumul	46099.	29431.	{29041., 29790.}	36.157	0.194908
8	newly 3 weeks spec	7809.	7715.9	{7598., 7826.}	1.31873	0.00577959	newly 3 weeks cumul	28877.	28460.	{28290., 28614.}	1.44395	0.00632028	newly 5 weeks spec	4404.	4534.58	{4449., 4618.}	2.97139	0.012672	newly 5 weeks cumul	39237.	38998.5	{38876., 39156.}	0.61544	0.00268281	newly 54 weeks cumul	50550.	51432.7	{51302., 51570.}	1.74627	0.00751757
9	newly 3 weeks spec	8491.	7003.62	{6903., 7175.}	17.5171	0.0840987	newly 3 weeks cumul	32010.	28785.2	{28624., 29130.}	10.0745	0.0462725	newly 5 weeks spec	4649.	3071.94	{3003., 3206.}	33.9226	0.18132	newly 5 weeks cumul	43099.	36596.	{36491., 37078.}	15.0885	0.0712942	newly 54 weeks cumul	54004.	41690.3	{41611., 42243.}	22.8015	0.112772
10	newly 3 weeks spec	3970.	4605.28	{4186., 4842.}	16.002	0.0638418	newly 3 weeks cumul	22883.	25108.9	{23895., 25822.}	9.72713	0.0400921	newly 5 weeks spec	1101.	1331.52	{1153., 1457.}	21.0336	0.0813192	newly 5 weeks cumul	26005.	28954.	{27352., 29968.}	11.3402	0.0463642	newly 58 weeks cumul	27241.	30394.6	{28466., 31489.}	11.5765	0.0472364
11	newly 3 weeks spec	6532.	5151.39	{5038., 5250.}	21.1361	0.103184	newly 3 weeks cumul	23257.	19581.8	{19357., 19805.}	15.8028	0.0747209	newly 5 weeks spec	3974.	2848.29	{2745., 2954.}	28.3269	0.144812	newly 5 weeks cumul	32341.	26324.6	{25968., 26610.}	18.6031	0.0894105	newly 52 weeks cumul	43101.	33167.6	{32708., 33528.}	23.0468	0.11379
12	newly 3 weeks spec	8024.	4813.6	{4683., 4929.}	40.01	0.222022	newly 3 weeks cumul	31244.	23062.9	{22716., 23439.}	26.1846	0.131885	newly 5 weeks spec	4154.	1675.28	{1585., 1757.}	59.6707	0.394775	newly 5 weeks cumul	41376.	27616.4	{27228., 28026.}	33.2551	0.175612	newly 53 weeks cumul	50863.	29786.7	{29403., 30271.}	41.4373	0.232408
13	newly 3 weeks spec	7496.	6125.38	{5987., 6273.}	18.2847	0.0877557	newly 3 weeks cumul	24982.	22312.3	{22048., 22574.}	10.6867	0.0491018	newly 5 weeks spec	4998.	3512.64	{3392., 3609.}	29.7191	0.153284	newly 5 weeks cumul	36270.	30529.4	{30211., 30874.}	15.8275	0.0748451	newly 51 weeks cumul	54152.	39037.	{38651., 39395.}	27.9121	0.14215
14	newly 3 weeks spec	5024.	5168.25	{4665., 5690.}	8.19049	0.0346365	newly 3 weeks cumul	30056.	30100.1	{28480., 31533.}	3.86545	0.0167761	newly 5 weeks spec	1153.	1157.68	{940., 1371.}	13.5854	0.0592614	newly 5 weeks cumul	33840.	33766.6	{31776., 35657.}	4.69681	0.0204538	newly 56 weeks cumul	34818.	34722.8	{32617., 36834.}	5.06525	0.0220716
15	newly 3 weeks spec	5483.	6067.82	{5920., 6185.}	10.6661	0.0439448	newly 3 weeks cumul	26035.	27781.9	{27505., 27999.}	6.70978	0.0281936	newly 5 weeks spec	1932.	2337.32	{2263., 2404.}	20.9793	0.0825845	newly 5 weeks cumul	31301.	33936.4	{33628., 34157.}	8.41957	0.0350996	newly 56 weeks cumul	33894.	37336.2	{37026., 37595.}	10.1557	0.0420005
16	newly 3 weeks spec	6616.	6388.25	{6268., 6529.}	3.44241	0.0152609	newly 3 weeks cumul	30832.	30309.3	{30110., 30492.}	1.71153	0.00750222	newly 5 weeks spec	2584.	2385.31	{2312., 2467.}	7.68924	0.0348698	newly 5 weeks cumul	37486.	36645.8	{36436., 36837.}	2.25647	0.00991583	newly 56 weeks cumul	41249.	40116.8	{39891., 40307.}	2.76749	0.0121915
17	newly 3 weeks spec	6952.	5698.97	{5589., 5825.}	18.024	0.086366	newly 3 weeks cumul	24739.	20969.1	{20696., 21220.}	15.2385	0.0718189	newly 5 weeks spec	4470.	3271.2	{3168., 3378.}	26.8188	0.135719	newly 5 weeks cumul	34818.	28628.	{28355., 28975.}	17.7782	0.0850282	newly 51 weeks cumul	48456.	36767.9	{36428., 37150.}	24.1211	0.119892
18	newly 3 weeks spec	7355.	7029.42	{6899., 7197.}	4.42665	0.0197175	newly 3 weeks cumul	26994.	25738.5	{25515., 25994.}	4.65103	0.0206973	newly 5 weeks spec	4020.	3815.43	{3715., 3917.}	5.08881	0.0227779	newly 5 weeks cumul	36463.	34843.5	{34519., 35149.}	4.44141	0.01974	newly 52 weeks cumul	45100.	43115.9	{42802., 43434.}	4.39938	0.0195466
19	newly 3 weeks spec	8310.	7885.17	{7779., 7997.}	5.11227	0.0228189	newly 3 weeks cumul	31919.	30516.7	{30308., 30691.}	4.39325	0.0195163	newly 5 weeks spec	4512.	4057.19	{3981., 4139.}	10.08	0.0461997	newly 5 weeks cumul	42585.	40312.7	{40124., 40486.}	5.33601	0.0238179	newly 54 weeks cumul	52753.	48802.3	{48586., 48993.}	7.48905	0.0338088
20	newly 3 weeks spec	6547.	5181.69	{5075., 5279.}	20.854	0.101626	newly 3 weeks cumul	38691.	35860.8	{35599., 36087.}	7.31501	0.0329974	newly 5 weeks spec	1542.	1013.07	{967., 1056.}	34.3016	0.182704	newly 5 weeks cumul	43499.	39198.3	{38959., 39412.}	9.88692	0.045217	newly 59 weeks cumul	44967.	39964.9	{39700., 40199.}	11.124	0.0512203
21	newly 3 weeks spec	7119.	6873.51	{6729., 6992.}	3.44838	0.0152898	newly 3 weeks cumul	29356.	29559.3	{29334., 29802.}	0.748126	0.00323074	newly 5 weeks spec	3172.	2830.36	{2749., 2899.}	10.7705	0.0495848	newly 5 weeks cumul	37313.	36898.3	{36625., 37092.}	1.11784	0.00488761	newly 56 weeks cumul	42719.	41302.7	{41011., 41587.}	3.31536	0.0146481
22	newly 3 weeks spec	6759.	7149.98	{7027., 7316.}	6.21571	0.0261696	newly 3 weeks cumul	29716.	30689.4	{30453., 31031.}	3.36704	0.0143653	newly 5 weeks spec	2827.	3019.19	{2923., 3139.}	8.25716	0.0346881	newly 5 weeks cumul	37047.	38432.2	{38271., 38864.}	4.05088	0.0172518	newly 57 weeks cumul	41620.	43362.7	{43269., 43837.}	4.75548	0.0202233
23	newly 3 weeks spec	6270.	6117.77	{5995., 6231.}	2.44833	0.0108043	newly 3 weeks cumul	22584.	22413.2	{22186., 22616.}	0.837628	0.00366076	newly 5 weeks spec	3912.	3606.43	{3520., 3693.}	7.81109	0.0354026	newly 5 weeks cumul	31596.	30778.	{30542., 31001.}	2.58881	0.0113989	newly 50 weeks cumul	43028.	40303.	{40092., 40535.}	6.33311	0.0284182
24	newly 3 weeks spec	7567.	5051.35	{4918., 5156.}	33.245	0.175583	newly 3 weeks cumul	27322.	21196.7	{20798., 21482.}	22.4189	0.110271	newly 5 weeks spec	4888.	2233.19	{2127., 2329.}	54.3128	0.340461	newly 5 weeks cumul	38440.	26844.4	{26459., 27175.}	30.1654	0.155951	newly 50 weeks cumul	52801.	30669.2	{30282., 31010.}	41.9155	0.235959
25	newly 3 weeks spec	6782.	5279.86	{5134., 5388.}	22.1489	0.108918	newly 3 weeks cumul	30207.	26906.3	{26593., 27198.}	10.9269	0.0502697	newly 5 weeks spec	2743.	1696.65	{1600., 1748.}	38.1462	0.209717	newly 5 weeks cumul	37399.	31639.6	{31257., 31951.}	15.4	0.0726694	newly 53 weeks cumul	41988.	33704.5	{33249., 33917.}	19.7283	0.0955123
26	newly 3 weeks spec	7069.	5516.54	{5386., 5624.}	21.9615	0.107755	newly 3 weeks cumul	23874.	20664.6	{20411., 20913.}	13.4432	0.0627199	newly 5 weeks spec	4701.	3052.59	{2946., 3139.}	35.0651	0.18766	newly 5 weeks cumul	34401.	27890.5	{27535., 28227.}	18.9255	0.0911339	newly 51 weeks cumul	50936.	35146.7	{34768., 35549.}	30.9983	0.161157
27	newly 3 weeks spec	6335.	5208.27	{5105., 5323.}	17.7858	0.0851101	newly 3 weeks cumul	22631.	20101.3	{19935., 20288.}	11.178	0.0514905	newly 5 weeks spec	3937.	2854.41	{2759., 2934.}	27.4978	0.139777	newly 5 weeks cumul	31837.	26856.6	{26631., 27042.}	15.6433	0.0738886	newly 52 weeks cumul	45079.	33826.8	{33502., 34159.}	24.961	0.124723
28	newly 3 weeks spec	7243.	5466.27	{5328., 5609.}	24.5303	0.122309	newly 3 weeks cumul	27640.	23585.3	{23303., 23874.}	14.6696	0.0689135	newly 5 weeks spec	4186.	2383.16	{2282., 2464.}	43.0683	0.24481	newly 5 weeks cumul	37414.	29626.1	{29292., 29915.}	20.8154	0.101375	newly 56 weeks cumul	48001.	33669.1	{33270., 34010.}	29.8575	0.154039
29	newly 3 weeks spec	6769.	5018.17	{4902., 5140.}	25.8654	0.130061	newly 3 weeks cumul	23489.	18999.	{18753., 19280.}	19.1153	0.0921616	newly 5 weeks spec	4398.	2741.23	{2636., 2838.}	37.671	0.205477	newly 5 weeks cumul	33395.	25511.1	{25139., 25882.}	23.6081	0.116979	newly 50 weeks cumul	46315.	31761.7	{31358., 32187.}	31.4225	0.163842
30	newly 3 weeks spec	8192.	5447.15	{5086., 6156.}	33.5065	0.178481	newly 3 weeks cumul	34290.	28415.7	{27494., 30428.}	17.1313	0.0819722	newly 5 weeks spec	3775.	1619.05	{1432., 1996.}	57.1113	0.371894	newly 5 weeks cumul	43617.	33042.2	{31827., 36039.}	24.2446	0.121164	newly 59 weeks cumul	50418.	34801.6	{33429., 38467.}	30.9739	0.161762
31	newly 3 weeks spec	7625.	6520.16	{6271., 6893.}	15.023	0.0709721	newly 3 weeks cumul	37142.	34555.6	{33800., 35854.}	7.24821	0.0327513	newly 5 weeks spec	2494.	1835.44	{1691., 2006.}	27.6784	0.141426	newly 5 weeks cumul	43977.	39921.8	{39038., 41588.}	9.63167	0.0440722	newly 60 weeks cumul	46875.	41843.6	{40979., 43742.}	11.2746	0.052029
32	newly 3 weeks spec	6240.	8132.27	{8021., 8254.}	30.3248	0.115001	newly 3 weeks cumul	27473.	31293.1	{31100., 31449.}	13.905	0.0565384	newly 5 weeks spec	2353.	4216.6	{4134., 4310.}	79.201	0.253278	newly 5 weeks cumul	33807.	41458.9	{41260., 41643.}	22.6341	0.0886081	newly 57 weeks cumul	37286.	50538.5	{50391., 50692.}	35.5429	0.132075
33	newly 3 weeks spec	8467.	7886.94	{7728., 8011.}	6.85083	0.0308524	newly 3 weeks cumul	28949.	28443.6	{28214., 28646.}	1.74569	0.00765558	newly 5 weeks spec	5209.	4515.58	{4425., 4615.}	13.312	0.0621055	newly 5 weeks cumul	40750.	39034.3	{38795., 39309.}	4.21021	0.0186859	newly 55 weeks cumul	56254.	49905.5	{49663., 50115.}	11.2854	0.052008
34	newly 3 weeks spec	6053.	6946.99	{6847., 7060.}	14.7694	0.0597917	newly 3 weeks cumul	19976.	22482.	{22315., 22642.}	12.5453	0.0513187	newly 5 weeks spec	4286.	5188.54	{5082., 5291.}	21.0579	0.082941	newly 5 weeks cumul	29425.	33761.	{33563., 33954.}	14.7358	0.0596946	newly 50 weeks cumul	43351.	55094.1	{54937., 55309.}	27.0883	0.104104
35	newly 3 weeks spec	8823.	8474.44	{8378., 8628.}	3.95058	0.0177161	newly 3 weeks cumul	31843.	31979.8	{31850., 32245.}	1.02428	0.00449606	newly 5 weeks spec	5272.	4374.08	{4282., 4510.}	17.0319	0.0818159	newly 5 weeks cumul	44053.	42572.	{42505., 42959.}	3.36181	0.0149886	newly 55 weeks cumul	57367.	51663.3	{51681., 52213.}	9.94246	0.0457512
36	newly 3 weeks spec	6496.	6820.04	{6726., 6913.}	4.9883	0.0211159	newly 3 weeks cumul	23327.	24438.4	{24266., 24601.}	4.76431	0.0202075	newly 5 weeks spec	4189.	4179.24	{4085., 4263.}	1.2953	0.0056411	newly 5 weeks cumul	32804.	34028.3	{33816., 34199.}	3.73204	0.0159087	newly 53 weeks cumul	44915.	45900.1	{45607., 46173.}	2.19325	0.00941825
37	newly 3 weeks spec	6929.	6998.48	{6841., 7146.}	2.0736	0.00910867	newly 3 weeks cumul	31730.	31720.3	{31513., 31986.}	0.675039	0.00299146	newly 5 weeks spec	2540.	2739.19	{2602., 2838.}	8.68228	0.0362469	newly 5 weeks cumul	38553.	38901.	{38456., 39183.}	1.40383	0.00614248	newly 58 weeks cumul	42395.	43028.1	{42232., 43333.}	2.10768	0.00916341
38	newly 3 weeks spec	5419.	4336.55	{4165., 4483.}	19.9751	0.09692	newly 3 weeks cumul	32641.	28898.4	{28521., 29336.}	11.4659	0.052916	newly 5 weeks spec	1229.	859.05	{787., 919.}	30.1017	0.156236	newly 5 weeks cumul	36650.	31716.8	{31362., 32112.}	13.4602	0.0628046	newly 58 weeks cumul	37768.	32362.4	{32016., 32754.}	14.3126	0.0671023
39	newly 3 weeks spec	5970.	5606.65	{5477., 5740.}	6.08626	0.0273411	newly 3 weeks cumul	21972.	21375.2	{21025., 21785.}	2.74759	0.0121351	newly 5 weeks spec	3152.	2857.63	{2759., 2972.}	9.33915	0.0427599	newly 5 weeks cumul	29579.	28308.6	{27919., 28765.}	4.29487	0.0190956	newly 52 weeks cumul	36448.	33987.3	{33481., 34422.}	6.75129	0.0303843
40	newly 3 weeks spec	6214.	5068.58	{4967., 5179.}	18.4329	0.0885483	newly 3 weeks cumul	22734.	19933.9	{19721., 20153.}	12.3167	0.0570995	newly 5 weeks spec	3630.	2626.64	{2528., 2712.}	27.6408	0.140655	newly 5 weeks cumul	31216.	26261.2	{26021., 26510.}	15.8727	0.0750772	newly 53 weeks cumul	41146.	31988.6	{31708., 32261.}	22.2559	0.109344
41	newly 3 weeks spec	5376.	5886.82	{5768., 6001.}	9.50186	0.0393604	newly 3 weeks cumul	21206.	22365.5	{22129., 22600.}	5.46789	0.0231023	newly 5 weeks spec	2830.	3144.86	{3044., 3256.}	11.1258	0.0456724	newly 5 weeks cumul	27896.	29874.7	{29603., 30200.}	7.09295	0.0297454	newly 54 weeks cumul	33607.	36840.	{36465., 37271.}	9.62008	0.0398752
42	newly 3 weeks spec	4668.	4823.43	{4665., 4932.}	3.41667	0.0145086	newly 3 weeks cumul	24350.	24769.	{24385., 25150.}	1.78049	0.00764146	newly 5 weeks spec	1451.	1484.23	{1403., 1571.}	3.9552	0.0167999	newly 5 weeks cumul	28470.	28977.4	{28595., 29384.}	1.82266	0.00782353	newly 58 weeks cumul	30111.	30652.3	{30252., 31044.}	1.82256	0.00782335
43	newly 3 weeks spec	8069.	6480.53	{6373., 6606.}	19.6861	0.0952491	newly 3 weeks cumul	29202.	25728.5	{25458., 25990.}	11.8949	0.0550133	newly 5 weeks spec	4750.	3077.8	{2975., 3165.}	35.2042	0.188585	newly 5 weeks cumul	40208.	33358.3	{33029., 33685.}	17.0358	0.0811198	newly 51 weeks cumul	53458.	39080.5	{38786., 39396.}	26.895	0.136062
44	newly 3 weeks spec	6354.	6218.09	{6143., 6394.}	2.42037	0.0111374	newly 3 weeks cumul	27932.	27800.3	{27735., 28060.}	0.833095	0.00374532	newly 5 weeks spec	2650.	2670.46	{2631., 2799.}	4.41962	0.0206447	newly 5 weeks cumul	34711.	34584.8	{34659., 34968.}	1.09818	0.00500898	newly 57 weeks cumul	39102.	39273.7	{39469., 39781.}	2.21705	0.00997733
45	newly 3 weeks spec	4797.	6154.12	{6046., 6274.}	28.291	0.108119	newly 3 weeks cumul	26104.	29434.1	{29265., 29609.}	12.7572	0.0521354	newly 5 weeks spec	1420.	2309.83	{2251., 2371.}	62.6641	0.211063	newly 5 weeks cumul	30202.	35551.7	{35393., 35734.}	17.7131	0.0708096	newly 56 weeks cumul	31840.	38885.1	{38728., 39107.}	22.1266	0.0867865
46	newly 3 weeks spec	8356.	7806.85	{6367., 8662.}	9.80242	0.0474234	newly 3 weeks cumul	33903.	32843.5	{29320., 34887.}	6.08542	0.0278055	newly 5 weeks spec	3539.	3236.01	{2151., 3854.}	18.1953	0.0934295	newly 5 weeks cumul	42756.	41188.1	{35221., 44445.}	8.29589	0.0383695	newly 56 weeks cumul	48435.	46297.4	{37888., 50857.}	10.4555	0.0491249
47	newly 3 weeks spec	6682.	6998.85	{6723., 7509.}	4.84122	0.0201367	newly 3 weeks cumul	30081.	30626.	{30003., 31818.}	1.95838	0.00831656	newly 5 weeks spec	2681.	2812.47	{2599., 3198.}	6.41589	0.0260186	newly 5 weeks cumul	37114.	37941.5	{36981., 39953.}	2.41507	0.0101662	newly 58 weeks cumul	40818.	42216.5	{40881., 45099.}	3.44968	0.0143615
48	newly 3 weeks spec	8702.	8015.8	{7591., 8250.}	7.88554	0.0358563	newly 3 weeks cumul	37680.	36822.2	{36078., 37219.}	2.27667	0.0100332	newly 5 weeks spec	3585.	2850.95	{2570., 3008.}	20.4756	0.100253	newly 5 weeks cumul	46811.	44562.5	{43262., 45163.}	4.80334	0.021442	newly 58 weeks cumul	52174.	48351.6	{46504., 49150.}	7.32635	0.0331519
49	newly 3 weeks spec	4127.	3345.63	{3243., 3470.}	18.9331	0.0915874	newly 3 weeks cumul	27465.	24225.9	{24010., 24545.}	11.7937	0.0546128	newly 5 weeks spec	921.	675.31	{627., 728.}	26.6764	0.13614	newly 5 weeks cumul	30359.	26413.4	{26218., 26805.}	12.9964	0.0605985	newly 60 weeks cumul	31206.	26947.	{26779., 27323.}	13.648	0.0638725
50	newly 3 weeks spec	6476.	4261.4	{4145., 4367.}	34.197	0.181845	newly 3 weeks cumul	23838.	18217.9	{17972., 18476.}	23.5761	0.116794	newly 5 weeks spec	3992.	1933.83	{1850., 2020.}	51.5574	0.315021	newly 5 weeks cumul	32964.	23052.8	{22733., 23331.}	30.0669	0.155339	newly 51 weeks cumul	44879.	26623.	{26246., 26980.}	40.6783	0.226809
